# Editorial: How Humans Recognize Objects: Segmentation, Categorization and Individual Identification

**DOI:** 10.3389/fpsyg.2016.00400

**Published:** 2016-03-22

**Authors:** Chris Fields

**Affiliations:** New Mexico State UniversityLas Cruces, NM, USA (Retired)

**Keywords:** space, time, object persistence, individual identity

What does it mean to say that something is an *object*? How do we recognize objects as such, picking them out from any non-objects that might happen to be present? What, indeed, does it mean to say that something is *not* an object? Is it even possible to recognize a non-object?

What, moreover, does it mean to say that something is a specific, *individual* object. Suppose you are handed 10 brand-new 1 € coins, each of which looks and feels exactly like the others. How do we recognize one of them as exactly the same individual 1 € coin we were looking at a moment ago? How does this process change if we've looked away for a few seconds, a minute, an hour? What if we have not seen the coin since last year? How does the individual recognition process change if, instead of coins, we are talking about 10 new colleagues encountered at a meeting 1 year ago?

The “what does it mean” versions of these questions have been with us since antiquity, in the form of philosophical musings about the nature of or evidence for an external world. The “how” versions have been asked for slightly over a century, and a detailed picture has begun to emerge only in the past two decades. Schneider's (1969) suggestion that two distinct pathways support visual orientation toward object features and locations was a watershed event in this growing understanding (see Goodale and Milner, 1992 for an early review). Research stemming from this idea has inextricably linked object recognition to the experiences of space, time, and persistence over time, i.e., individual identity (see Scholl, 2007; Fields, 2012 for review). Without a spacetime “container” and individual, time-persistent objects, motion and causation cannot be defined; hence object recognition underlies these experiences as well.

The papers in this Research Topic provide a glimpse of the current state of understanding the “how” of object recognition. Beginning with the most concrete, Taylor et al. review the development of contour detection and integration in humans, relating the functional trajectory from infancy to adolescence to the increasing range of horizontal connectivity within areas V1 and V2 during the same period. Kosilo et al. then describe new experiments designed to tease apart the effects of low-level (color and contrast) and high-level (identifiability as an object) stimulus features on the control of visual saccades. Schendan and Ganis show that object recognition exerts top-down effects on visual processing within 250 ms; Caplette et al. demonstrate the influence of top-down affective and contextual expectations on the precision with which objects are represented. Anzellotti and Caramazza review evidence suggesting that human face identity is selectively encoded in the right-hemisphere anterior temporal pole (ATP), an area generally implicated in semantic memory. Orban et al. review the functional anatomy of the ventral stream, and suggest that fully-defined individual entities of all types are represented in ATP.

Lacey and Sathian review visuo-haptic integration, focusing on the role of lateral occipital cortex (LOC); Kassuba et al. describe downstream effects on visual and haptic processing following disruption of LOC activity by transcranial magnetic stimulation. Maranesi et al. review the representation of motor affordances and their activation by object recognition, while Schubotz et al. present new results on the representation of action expectations. Schlesinger et al. address the key question of how infants learn to generate expectations that predict the behavior of the visual world.

The remaining five papers address fundamental theoretical issues. Grossberg et al. address the question of scene stability across eye movements using the Adaptive Resonance Theory framework. Bruza and Chang investigate the utility of quantum probabilities for explaining relevance judgments. Aerts reviews quantum theory itself, explaining why it renders the existence of the separate, bounded entities that we call “objects” mysterious. Klein examines the human perception of a time-persistence self and suggests that sameness is a pre-evidential “default mode” of the self representation. Hoffman and Prakash review evidence suggesting that neither objects nor their spacetime “container” objectively exist, but must instead be considered to be emergent from multi-agent interactions.

Beyond the leading edge represented by these papers lie questions for further research, many of which concern the development, especially during early infancy, of object-recognition capabilities. Three of the most significant, in my opinion, are the following.

1. How malleable are the human representations of space and time? Are particular motor capabilities essential to the development of these representations? What is the role of sensory-motor correlations in representing perceived space? Would an organism inhabiting a world devoid of manipulable objects be able to develop a 3d spatial representation?
  Recent developments in quantum theory have led to a new emphasis among physicists on reference frames as physical objects, not just abstract coordinate systems, with respect to which quantities are measured: examples include clocks and gyroscopes used as reference frames to measure time and spatial orientation, respectively (Bartlett et al., 2007). What are the earliest-developing reference frames for space and time in humans? By what age do infants perceive objects as embedded in a containing space that imposes relationships upon them, as opposed to just perceiving objects?
2. How do causal reasoning and object recognition ability co-develop? Is there some particular level of predictability that is required? What kind of predictability—predictable locations or motions, predictable static features, or both? What would happen in an environment in which the predictability of locations and motions was uncorrelated with the predictability of static features?
  Any object that serves as a reference frame must be unproblematically recognizable as such: a clock, for example, can only serve as a clock if its identity over time is not in question. What level of predictability must the infant environment have in order for typical space and time reference frames to develop? What level of predictability must it have in order for typical object categories to develop? What happens in environments with less than this critical level of predictability?
3. How does the subjectively-accessible sense of the body as a time-persistent object and hence of the stably-embodied self develop? Rochat (2012) suggests that a rudimentary embodied-self representation is present at birth. How is this representation implemented? How is this implementation constructed prenatally?
  If Hoffman and Prakash are right in stating that a shared external world of objectively-defined objects cannot be assumed, the infant's representation of itself and its capabilities for action becomes the only reference frame from which a perceived world of persistent objects can be constructed. What level of coherence must the world provide, whatever its structure, for this process of construction to be feasible?

These questions cannot, clearly, be fully answered by experiments with human infants. Combining experiments that are feasible with infants with experiments carried out on validated computational models, as in the work of Schlesinger et al. promises to become even more important as questions such as those contemplated here are addressed.

## Author contributions

The author confirms being the sole contributor of this work and approved it for publication.

### Conflict of interest statement

The author declares that the research was conducted in the absence of any commercial or financial relationships that could be construed as a potential conflict of interest.
